# Context-dependent roles of UCP2 in cancer: functional heterogeneity, molecular mechanisms and translational perspectives

**DOI:** 10.3389/fonc.2026.1906646

**Published:** 2026-08-10

**Authors:** Wangde Jin, Xinglin Jin

**Affiliations:** Department of Hepatobiliary Surgery, Yanbian University Hospital, Yanji, Jilin, China

**Keywords:** functional heterogeneity, mitochondrial metabolism, redox homeostasis, translational medicine, tumor immune microenvironment, tumor metabolic reprogramming, UCP2

## Abstract

Uncoupling protein 2 (UCP2), a member of the solute carrier family 25 (SLC25) of mitochondrial carriers, is situated in the inner mitochondrial membrane and has conventionally been associated with proton leak, mitochondrial membrane potential, and control of reactive oxygen species (ROS). Recent research has expanded this perspective by establishing connections between UCP2 and C4 metabolite transport, glutamine utilization, tricarboxylic acid (TCA) cycle anaplerosis, and metabolic restructuring of the tumor immune microenvironment. Thus, the significance of UCP2 in cancer surpasses the traditional role of an energy-dissipating protein. Present data suggest that UCP2 exhibits context-specific roles across various cancer types. In certain contexts, it facilitates tumor adaptation by preserving redox balance, supporting glutamine metabolism, and enhancing metabolic flexibility under therapeutic pressure. Conversely, in different cancer types or immune-cell environments, UCP2 expression correlates with unique prognostic trends, treatment outcomes, or immune infiltration characteristics. This review outlines UCP2 biology with a focus on context specificity, highlighting its expression patterns across different cancers, metabolic alterations, redox regulation, intercellular signaling, and the tumor immune milieu. Furthermore, we explore the translational implications of UCP2 as both a biomarker and a therapeutic target. In essence, UCP2 should be regarded as a metabolic vulnerability that necessitates classification based on cancer type, mutational profile, metabolic requirements, and immune status, rather than as a universal target for broad-spectrum cancer inhibition.

## 1 Introduction

Tumorigenesis and cancer progression are driven not only by the gradual accumulation of genetic alterations but also by persistent metabolic reprogramming (1). While the Warburg effect has historically influenced the understanding of cancer metabolism, emerging evidence indicates that mitochondria in cancer cells are not merely inactive. Rather, they undergo reconfiguration in response to specific genetic and microenvironmental pressures, facilitating energy production, biosynthesis, redox buffering, and immune evasion (2–5). In this context, inner mitochondrial membrane proteins serve as active components of the metabolic network. Their functional state may play a crucial role in determining whether cancer cells can sustain a survival advantage under conditions of hypoxia, nutrient deprivation, and therapeutic stress (3, 4).

UCP2 is a highly debated mitochondrial regulator with potential translational implications in cancer. Traditionally, UCP2 has been viewed as an agent that exerts antioxidant and metabolic protective effects by promoting proton leak, decreasing mitochondrial membrane potential, and limiting excessive mitochondrial ROS production across various cell types (6–8). However, recent years have seen a redefinition of its structural and metabolic roles. UCP2 is now recognized for its involvement in C4 metabolite transport, glutamine-driven TCA cycle anaplerosis, and aspartate export. In the context of KRAS-driven pancreatic cancer, for instance, UCP2-mediated aspartate export connects mitochondrial metabolism to cytosolic metabolic networks (9–11). Consequently, the importance of UCP2 in cancer should not be confined to its role in promoting uncoupling; rather, it must be assessed within the broader context of metabolic flux, redox thresholds, nutrient utilization, and cell-fate decisions.

Conversely, in breast cancer, specific NSCLC models, and immune-cell contexts, the relationship between UCP2 expression, prognosis, treatment response, and antitumor immunity is not straightforward (12–15). In this framework, context dependence signifies that the biological effects of UCP2 are influenced not solely by expression levels but also by cancer lineage, driver mutations, substrate reliance, redox thresholds, immune-cell composition, and treatment pressures.

Here, context specificity denotes more than variable expression; it refers to the fact that UCP2 function is conditioned by at least four interacting dimensions: cancer lineage, driver-mutation background, metabolic dependency, and immune/stromal state. This framework helps explain why the same change in UCP2 may support survival in glutamine-addicted or high-ROS tumors, appear mainly correlative in other settings, or even generate competing consequences when tumor cells and immune cells are considered together. Accordingly, this review is organized around biologically defined contexts rather than around a single universal model of UCP2 action.

## 2 The fundamental biology of UCP2: from classical uncoupling to metabolite transport

### 2.1 Molecular structure, localization, and family features

UCP2 is a member of the SLC25 mitochondrial carrier superfamily and resides predominantly in the inner mitochondrial membrane. Along with UCP1 and UCP3, it belongs to the uncoupling protein family (6–8). Superficial similarity to UCP1 can be misleading: UCP1 is specialized for brown-fat thermogenesis, while UCP2 displays broader tissue distribution and condition-dependent protein expression. Although UCP2 mRNA is detectable across many organs, protein abundance is more tightly regulated. Elevated UCP2 protein levels are typically observed in cells subject to high metabolic or stress demands, including immune cells, pancreas, lung, intestine, white adipose tissue, and several tumor models (6). This distribution implies an inducible mitochondrial regulator rather than a constitutive thermogenic uncoupler. In cancer, UCP2’s inner-membrane localization situates it close to oxidative phosphorylation, reactive oxygen species generation, metabolite exchange, and cell-death signaling (7).

### 2.2 Classical functions: proton leak, membrane potential, and ROS regulation

The classical model proposes that UCP2 permits mild proton re-entry from the intermembrane space into the matrix, partially lowering the mitochondrial membrane potential (delta-psi m). When electron supply exceeds ATP demand, complexes I and III remain highly reduced and are more prone to superoxide formation; a small decrease in delta-psi m is therefore expected to relieve electron back-pressure and decrease ROS production. This framework explains why increased UCP2 is often associated with decreased oxidative stress, improved stress tolerance, and lower apoptosis in some cancer models (6–8). However, the cancer literature does not support a uniform interpretation. In several settings, the apparent ROS-lowering effect of UCP2 may also reflect altered substrate flux, C4 metabolite export, calcium handling, or glutathione-dependent buffering rather than direct proton conductance alone (7, 8, 11). Thus, UCP2 is better viewed as a regulator of the redox operating range of mitochondria than as a simple antioxidant protein.

### 2.3 Expanded functions: C4 metabolite transport and glutamine-dependent metabolism

Recent work has redefined UCP2 from a presumed proton-leak protein to a transporter that can exchange C4 metabolites. In proteoliposome assays, Vozza et al. showed that UCP2 mediates export of malate, oxaloacetate, and aspartate in exchange with phosphate and proton equivalents, providing a mechanistic basis for linking UCP2 to mitochondrial-cytosolic carbon redistribution (11). This model has major implications for cancer metabolism. By altering the availability of C4 intermediates, UCP2 can influence whether carbon is retained within the mitochondrial TCA cycle or redirected toward cytosolic biosynthetic and redox-supporting pathways. In that sense, UCP2 may affect not only membrane potential but also the relative contribution of glutamine and glucose to anaplerosis, NADPH maintenance, and metabolic plasticity under stress (7, 8, 11).

Cancer models provide functional support for a transporter-centered view of UCP2. In KRAS-mutant PDAC, UCP2 mediates export of glutamine-derived aspartate into the GOT1-MDH1-ME1 pathway, thereby increasing NADPH production and maintaining the GSH/GSSG balance. UCP2 knockdown decreases glutaminolysis, lowers NADPH/NADP+ and GSH/GSSG ratios, raises ROS, and suppresses tumor progression (10). T-ALL displays a related but distinct pattern: UCP2 sustains glutamine-fueled TCA cycle activity and lipid synthesis through malate export, and its suppression causes oxidative metabolism-dependent cells to lose respiratory capacity, shift toward glycolysis, and proliferate less effectively (16). These findings extend UCP2’s role beyond a simple antioxidant function to include contributions to energy production, substrate utilization, biosynthesis, and redox regulation. The functional impact of UCP2 should not be inferred solely from its expression level; when UCP2 acts primarily as a metabolite transporter in a given context, its significance should be interpreted together with glutamine dependence, oxygen consumption rate (OCR), extracellular acidification rate (ECAR), TCA intermediate levels, NADPH/GSH status, and baseline ROS (6, 9–11, 16, 17).

### 2.4 Major controversies in UCP2 biology

**Figure 1:**
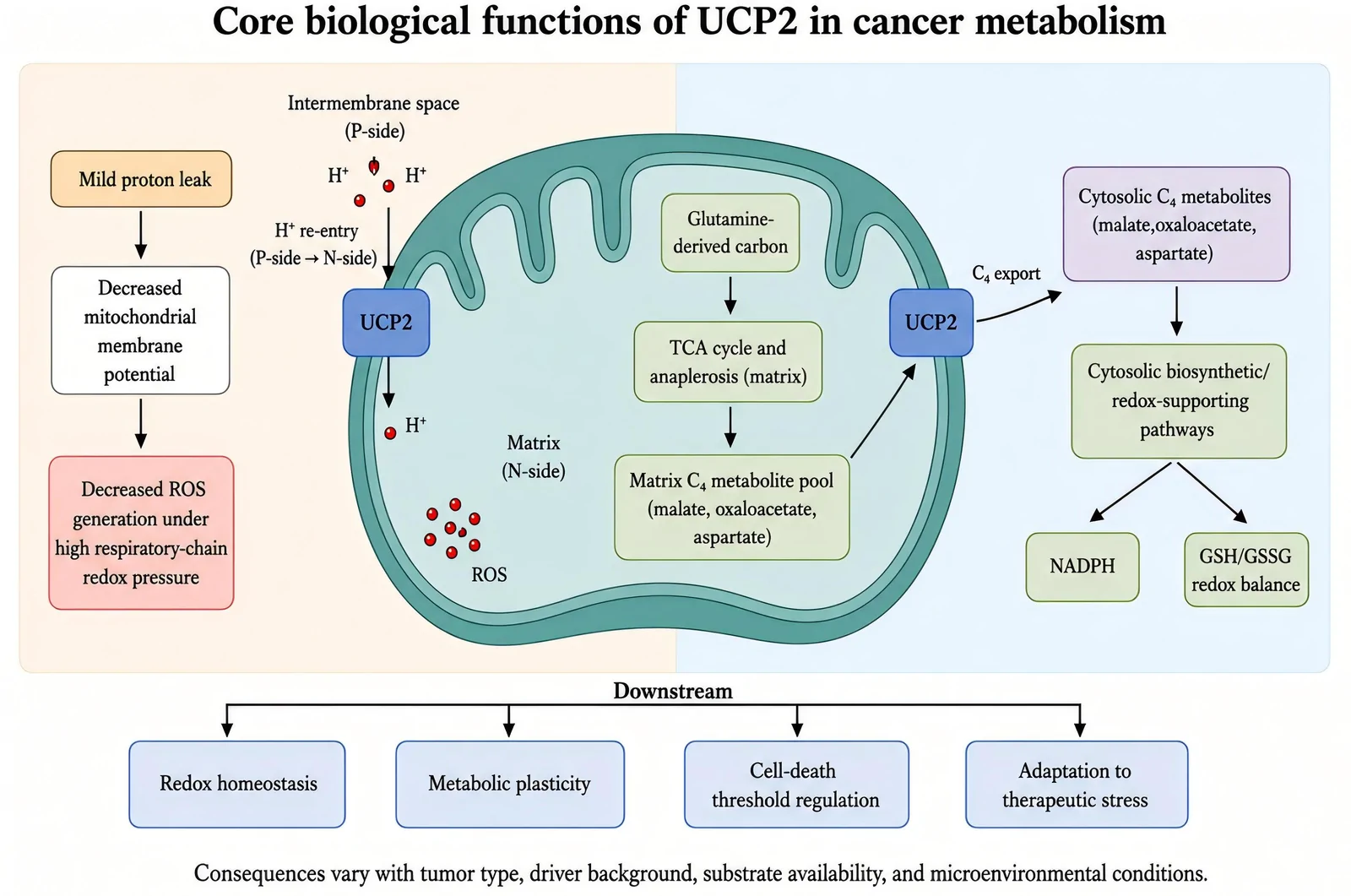
Proposed roles of UCP2 in redox control and carbon handling in cancer cells. UCP2 is located in the inner mitochondrial membrane between the intermembrane space (P-side) and the matrix (N-side). In the proton-leak model, H+ re-enters the matrix through UCP2. This modest leak partially decreases the mitochondrial membrane potential and can limit ROS generation when the respiratory chain is highly reduced. The transport model depicts glutamine-derived carbon entering matrix TCA-cycle and anaplerotic reactions, which contribute to a matrix C4-metabolite pool. UCP2 then exports C4 metabolites, including malate, oxaloacetate, and aspartate, to the cytosol, where they may support biosynthetic pathways and redox homeostasis. Reverse C4 transport may occur under specific metabolic conditions, but it is not shown here as a general cancer mechanism. The relative importance of these effects is likely to depend on tumor type, driver background, substrate availability, and the microenvironment.

The central controversy in UCP2 biology revolves around whether its primary function should be characterized as mild uncoupling or mitochondrial metabolite transport. The former model highlights decreased ROS through a lower membrane potential, whereas the latter emphasizes remodeling of glucose oxidation, glutaminolysis, and cytosolic reducing-power generation through C4 metabolite export (**Figure 1**). These models are not mutually exclusive: C4 metabolite export can decrease the matrix supply of reducing equivalents to the respiratory chain and thereby indirectly decrease membrane potential and ROS production (7, 11). Consequently, the antioxidant effects attributed to UCP2 may stem from proton leak, metabolic flux redistribution, or a combination of both mechanisms. Given the tight coupling of membrane potential, redox state, and metabolite flow, UCP2 may exhibit different dominant functions in different cellular contexts. This functional plasticity explains why the same protein can yield opposing biological outcomes across different cancer types (9–11, 16).

### 2.5 Limitations of quantitative readouts, transport kinetics, and structural evidence

Distinguishing uncoupling from proton leak is essential. Traditionally, uncoupling denotes the re-entry of protons across the inner mitochondrial membrane that lowers the efficiency of oxidative phosphorylation. By contrast, proton leak is only one mechanism that can decrease membrane potential. In the case of UCP2, declines in membrane potential can also arise from metabolite transport, altered substrate oxidation, or changes in the load on the electron transport chain. Without concurrent measurements of oxygen consumption, ATP production, membrane potential, and metabolic flux, a drop in ROS or a change in JC-1/TMRE fluorescence is insufficient to conclude that UCP2 functions as an uncoupler (7, 8, 11). Most cancer studies still rely on relative readouts. Membrane potential is typically estimated with JC-1, TMRE, TMRM, or flow-cytometry signals, and ROS is often reported as a fold change in fluorescent-probe intensity. These assays are sensitive to dye loading, mitochondrial mass, gating strategy, and cell-line differences. Consequently, the field lacks a practical cutoff between mild and strong uncoupling, and it is often unclear whether a reported UCP2 effect corresponds to a physiologically meaningful millivolt-scale change in membrane potential. Although structural work on UCP1 and the mitochondrial ADP/ATP carrier has improved the broader carrier framework (18, 19), equivalent UCP2-specific structural evidence remains limited. Whenever possible, future studies should pair relative fluorescence readouts with oxygen consumption, ATP production, mitochondrial mass, and calibrated ROS measurements.

Accordingly, UCP2 is better characterized as an inner mitochondrial membrane carrier that combines uncoupling-related effects with metabolite transport functions rather than functioning solely as a UCP1-like thermogenic uncoupler.

## 3 Expression patterns and functional heterogeneity of UCP2 across cancer types

### 3.1 UCP2 in hematological malignancies: represented by AML and T-ALL

In hematological malignancies, the importance of UCP2 appears to depend on the metabolic state of the leukemia cell rather than on lineage alone. In T-cell acute lymphoblastic leukemia (T-ALL), UCP2 silencing preferentially impairs glutamine-dependent subsets, decreases entry of glutamine-derived carbon into the TCA cycle, shifts cells toward glycolytic compensation, and restrains proliferation (16). This pattern argues that UCP2 is not a universal proliferation switch in leukemia; instead, it helps maintain a metabolic program that is especially valuable when oxidative glutamine use is required. Evidence in acute myeloid leukemia (AML) further supports this context dependence. In AML models, UCP2 inhibition increases branched-chain-amino-acid-associated oxidative stress and is accompanied by altered PI3K/AKT/mTOR signaling and weaker leukemia-related phenotypes (20). Together, these data suggest that the functional significance of UCP2 in hematological malignancies is highest when leukemia cells rely on mitochondrial anaplerosis, redox buffering, and stress adaptation rather than when UCP2 is viewed only as a static expression marker (6, 16, 20).

### 3.2 UCP2 in solid tumors: stronger background dependence

In contrast to hematological malignancies, interpretation of UCP2 in solid tumors is even more dependent on tumor-specific metabolic context. Pancreatic ductal adenocarcinoma (PDAC) is the clearest example because KRAS-driven tumors rely heavily on glutamine anaplerosis, mitochondrial metabolic control, and redox maintenance. In this setting, UCP2-linked aspartate export can connect mitochondrial carbon flow to cytosolic biosynthetic demand and adaptive stress responses (10, 21, 22). Other solid tumors require more caution. In uterine leiomyosarcoma (ULMS), UCP2 is elevated in some specimens, and UCP2-targeted intervention increases ROS and suppresses tumor-associated phenotypes (23). In melanoma, UCP2 upregulation has been associated with Akt/mTOR- and ERK-related growth signaling (24). Pan-cancer and glioma-focused analyses likewise show that UCP2 expression, prognostic impact, and therapeutic relevance vary by tumor type (14). Taken together, these data suggest that UCP2 is most likely to confer advantage in solid tumors facing high metabolic stress and strong redox-control demands, but that conclusion cannot be generalized to all tumor lineages.

Pediatric solid tumors exemplify this challenge. Studies of neuroblastoma have linked metabolic reprogramming, fatty acid utilization, and the UCP2/PPAR-gamma axis (25, 26). However, direct functional evidence in these tumors is less robust than that available for PDAC, leukemia, or some lung cancer models. Thus, in this context UCP2 is more accurately characterized as a metabolism-associated factor than as definitive evidence of a pan-cancer tumor-promoting role.

### 3.3 Opposing conclusions from non-small cell lung cancer and breast cancer

NSCLC exemplifies this heterogeneity. In some NSCLC models, UCP2 drives proliferation and glycolysis via mTOR/HIF-1alpha signaling (27). In A549 cells, however, UCP2 is more closely linked to energy allocation and substrate utilization than to proliferation alone (13). Other NSCLC studies emphasize dependence on molecular context. UCP2-associated metabolic plasticity involving DDX5/UBAP2L, and LKB1/STK11-linked sensitivity to mitochondrial uncoupling, indicate that driver context alters how UCP2 biology is manifested (28, 29). Thus, in lung cancer UCP2 may promote glycolysis and growth in one setting while primarily marking metabolic adaptation in another. Breast cancer data further argue against a universal pro-tumor role. High UCP2 expression correlates with better prognosis and distinct immune infiltration patterns in breast cancer cohorts (12). That observation does not establish a protective mechanism, but it does contradict the notion that elevated UCP2 invariably signals greater malignancy. A cautious interpretation is that UCP2 levels in some breast tumors reflect mitochondrial state, redox balance, or immune context rather than direct invasion-promoting activity. Direct evidence that UCP2 maintains a more complete mitochondrial program in breast cancer remains limited (6, 12).

### 3.4 An explanatory framework for cross-cancer heterogeneity

Differences in UCP2 expression and function are unlikely to arise from a single mechanism (**Table 1**). The revised table now distinguishes not only functional direction but also the level of evidence supporting each interpretation, because mechanistic confidence varies substantially across tumor contexts. A more useful classification framework includes at least four interacting layers. First, lineage-defined context matters because tumors differ in baseline reliance on oxidative phosphorylation, glutamine anaplerosis, lipid oxidation, and ROS buffering. Second, mutation-defined context matters because drivers such as KRAS, LKB1/STK11, and PI3K/AKT/mTOR pathway alterations reshape mitochondrial flux and determine whether UCP2 is functionally required or merely co-regulated (10, 20, 27–29). Third, nutrient and redox context matters because oxygen availability, glutamine supply, and therapy-induced stress influence whether UCP2-mediated control of metabolite exchange or membrane potential becomes advantageous. Fourth, immune/stromal context matters because the same UCP2-dependent process may support tumor-cell survival while exerting different consequences in T cells or other microenvironmental compartments (15, 30–32). For these reasons, UCP2 should not be labeled categorically as either pro-cancer or tumor-suppressive. Its biological meaning must be interpreted within a defined context rather than inferred from expression alone.

**Table 1:** Expression trends, functional directions, and key mechanisms or explanations of UCP2 in different tumors.

Tumor type	Expression/functional trends	Functional direction	Key mechanisms or explanations	Main references	Level of evidence
AML	UCP2 supports leukemia-related phenotypes in selected AML models	Pro-tumor tendency	Branched-chain-amino-acid-associated oxidative stress and coupling with PI3K/AKT/mTOR signaling	(20)	Cell line + animal model
T-ALL	Glutamine-dependent leukemia subsets show stronger dependence on UCP2	Pro-tumor tendency	Glutamine anaplerosis, TCA-cycle remodeling, and maintenance of proliferative fitness	(16)	Cell line/metabolic profiling
PDAC	Supports KRAS-associated glutamine/aspartate metabolism	Context-dependent; therapeutic sensitization possible	C4 metabolite transport, redox support, ROS-GAPDH signaling, and mTOR-related combination vulnerability	(10, 21, 46, 47)	Cell line + animal model
ULMS	UCP2 is elevated in some tumor tissues and targeting it increases oxidative stress	Pro-tumor tendency	ROS buffering and mitochondrial stress adaptation	(23)	Patient tissue + preclinical models
Melanoma	UCP2 is upregulated and associated with growth-promoting signaling	Pro-tumor tendency	Activation of Akt/mTOR and ERK-related signaling	(24)	Cell line
NSCLC	Conclusions vary across model systems and driver backgrounds	Context-dependent	mTOR/HIF-1alpha signaling, DDX5/UBAP2L-linked plasticity, and LKB1/STK11 background	(13, 27–29)	Cell line + mechanistic studies
Breast cancer	High UCP2 expression correlates with better prognosis in some cohorts	Correlative/undetermined	May reflect immune infiltration or mitochondrial state, but direct causal evidence remains limited	(12, 37)	Patient cohort + cell line
Gallbladder cancer	UCP2 promotes proliferation and chemotherapy tolerance	Pro-tumor tendency	NF-kappaB/beta-catenin signaling and mitochondrial ROS regulation	(38)	Cell line + xenograft
Glioma/glioblastoma	Pan-cancer and model-based studies support a potentially targetable role for UCP2	Pro-tumor tendency; targetable context	Metabolic adaptation and ferroptosis-related vulnerability	(14, 39)	Patient dataset + experimental validation

Pro-tumor tendency means that higher UCP2 expression or activity mainly supports proliferation, survival, metabolic adaptation, or therapy tolerance in the cited model. Context-dependent means that the effect changes across cell lines, driver mutations, or metabolic states. Correlative/undetermined means that the evidence is mainly associative and does not yet prove a direct causal role. The level-of-evidence column indicates whether the conclusion is supported mainly by cell-line work, animal models, patient material, or combined evidence.

### 3.5 Molecular basis and evidence integration of context dependence

Expression data alone are insufficient to establish context dependence. The field must also explain how specific biochemical settings convert UCP2 activity into different phenotypes. Four mechanistic issues are especially important. First, the balance between mild uncoupling and C4 metabolite export is likely not fixed across tumors; the dominant consequence may depend on substrate supply, respiratory-chain load, and partner transporters (7, 8, 11). Second, post-translational regulation remains an underdeveloped but potentially important layer of control. Phosphorylation, oxidation, ubiquitination, and protein-interaction-dependent regulation are all plausible mechanisms by which UCP2 activity, stability, or carrier behavior could change without a large change in mRNA abundance, thereby contributing to discordance between expression and phenotype (6–9, 28). However, the field still lacks a validated map of cancer-relevant UCP2 modification sites, the enzymes responsible for installing or removing these modifications, and direct functional studies linking specific modifications to transport activity, half-life, or subcellular distribution. At present, post-translational regulation is therefore best regarded not as a fully established explanation, but as a critical knowledge gap that may account for context-dependent differences in UCP2 behavior. Third, UCP2 operates within a broader mitochondrial network that includes electron transport chain complexes, adenine nucleotide transporters, calcium-handling systems, and redox buffers; variation in that network can change how a similar UCP2 perturbation is phenotypically expressed. Fourth, tissue-level interpretation is limited when studies rely only on bulk expression because the measured signal may derive from tumor cells, infiltrating immune cells, or stromal elements in different proportions. Thus, future studies will need flux analysis, cell-type-resolved perturbation, and spatial or single-cell approaches to connect UCP2 expression with true biochemical activity in context (33–35).

## 4 The molecular mechanism by which UCP2 drives cancer phenotypes

### 4.1 Redox homeostasis and cell-death threshold

Redox regulation recurs throughout UCP2 biology, but the mechanistic question is not simply whether UCP2 lowers ROS. When NADH and FADH2 supply outpaces ATP use, a high membrane potential favors electron back-pressure at the respiratory chain, especially at complexes I and III, and thereby increases superoxide formation. Under those conditions, UCP2 may help keep ROS within a signaling-permissive range either by slightly decreasing delta-psi m or by redistributing carbon flux in a way that decreases matrix reducing pressure (6–8, 11). The most informative cancer examples are stress-dependent rather than constitutive. In uterine leiomyosarcoma (ULMS), UCP2 inhibition increases ROS and suppresses malignant phenotypes, supporting a survival role under oxidative pressure (23). In AML, UCP2 inhibition amplifies branched-chain-amino-acid-associated oxidative stress and weakens leukemia progression (20). In melanoma, elevated UCP2 accompanies Akt/mTOR- and ERK-related growth signaling, again consistent with a stress-adaptation role rather than a stand-alone oncogenic driver (24). Together, these models indicate that UCP2 often helps maintain a redox threshold compatible with survival, but the biological consequence depends on the surrounding metabolic program.

In many tumors, UCP2 is more accurately viewed as a stress-adaptation factor than as a direct driver of proliferation. Its primary role may be to modulate the redox state and raise the cell-death threshold during nutrient stress or therapeutic pressure. This interpretation has limitations, however. ROS effects are dose-dependent: moderate ROS support signaling, whereas excessive mitochondrial inhibition or uncoupling can induce an energy deficit and reveal metabolic vulnerabilities (6, 7, 27, 29). Recent cancer cell-line data reinforce this distinction. UCP2 expression and regulation vary substantially across cancer types and culture conditions (9). Thus, UCP2 is most appropriately characterized as a conditional regulator of redox homeostasis and death threshold rather than as a universal antioxidant oncogene.

### 4.2 Glutamine metabolism, aspartate/malate transport, and metabolic plasticity

If redox control explains why UCP2 can raise the cell-death threshold, the transport model explains why its importance becomes greatest in tumors with defined nutrient dependence. UCP2 is now better understood as a regulator of mitochondrial-cytosolic C4 metabolite exchange rather than as a simple heat-dissipating protein (9–11). This point is most clearly developed in pancreatic ductal adenocarcinoma (PDAC) and T-cell acute lymphoblastic leukemia (T-ALL). In KRAS-driven PDAC, UCP2-linked aspartate export has been proposed to support glutamine-dependent carbon flow, maintain cytosolic biosynthetic and redox demands, and thereby sustain tumor growth under metabolic stress (10, 21). In T-ALL, UCP2 silencing decreases oxidative use of glutamine-derived carbon, causes a compensatory glycolytic shift, and decreases proliferative fitness (16). These findings suggest that UCP2 does not simply turn one pathway on or off. Rather, it helps preserve continuity between mitochondrial anaplerosis, cytosolic biosynthesis, and redox buffering when cells are challenged by nutrient limitation or therapy. This is why UCP2 vulnerability is expected to be strongest in tumors that are highly dependent on glutamine utilization, mitochondrial anaplerosis, or redox buffering, and much weaker in tumors that can readily compensate through alternative metabolic routes (6, 10, 16, 17, 21, 36).

### 4.3 Coupling with PI3K/AKT/mTOR, HIF-1alpha, and LKB1/STK11-related signaling

The functional output of UCP2 extends beyond mitochondria alone and must be interpreted in the signaling background of each tumor. The same change in UCP2 expression may not have the same consequence in different driver contexts because metabolic stress and growth signaling are tightly coupled. In NSCLC, one study reported that UCP2 promotes proliferation and glycolysis through the mTOR/HIF-1alpha axis, suggesting that UCP2 can link mitochondrial adaptation to glycolytic reinforcement in a subset of lung cancer models (27). Other work in lung cancer emphasizes a different layer of context: UCP2-associated metabolic plasticity involving DDX5/UBAP2L and the altered sensitivity of LKB1/STK11-deficient cells to mitochondrial perturbation imply that UCP2-associated phenotypes are filtered through broader driver-defined networks rather than through UCP2 abundance alone (28, 29). In AML, the phenotype associated with UCP2 inhibition is again coupled to altered PI3K/AKT/mTOR signaling under oxidative stress (20). Thus, UCP2 should not be treated as an isolated signaling molecule. Its phenotypic effect is better interpreted as the output of an interaction between mitochondrial flux control, redox pressure, and oncogenic signaling architecture.

### 4.4 Conditional regulation of proliferation, therapy resistance, and invasiveness by UCP2

UCP2 influences proliferation, therapy response, and invasive behavior, but the strength of evidence differs markedly across models. In settings with high mitochondrial or redox stress, increased UCP2 activity can raise tolerance to chemotherapy, radiotherapy, or metabolic challenge by stabilizing redox balance and preserving metabolic plasticity (6, 20, 24, 27). In AML and ULMS, UCP2 inhibition increases oxidative injury and suppresses tumor-associated phenotypes (20, 23). In HER2-positive breast cancer cells, trastuzumab induces UCP2 expression, and genipin enhances trastuzumab-associated growth inhibition and apoptosis in BT474 cells, supporting the idea that UCP2 can participate in adaptive treatment resistance rather than constitutively drive growth (37). In gallbladder cancer, UCP2 promotes proliferation and chemotherapy tolerance through NF-kappaB/beta-catenin signaling and mitochondrial ROS control; its inhibition increases sensitivity to gemcitabine-like treatment (38). In glioblastoma, pharmacological targeting of UCP2 has been linked to ferroptosis-associated changes, including decreased GPX4, increased ACSL4, and attenuation of malignant phenotypes (39, 40). Taken together, these studies support UCP2 as a context-dependent sensitization node, but they do not justify a universal claim that UCP2 is a stable marker of invasion or drug resistance across cancers.

UCP2 cannot be simply characterized as a stable marker of drug resistance or invasion. Research on breast cancer prognosis indicates that high UCP2 expression is occasionally linked to improved clinical outcomes and specific immune infiltration characteristics (12). Additionally, immune-related studies suggest that the role of UCP2 in tumor cells may differ from its role in immune cells (15, 31, 32). Consequently, the phenotypic significance of UCP2 is evidently conditional: in tumors that rely heavily on redox buffering and mitochondrial metabolic adaptation, UCP2 may contribute to therapeutic tolerance. In other contexts, its expression is more likely to indicate mitochondrial functional status, immune-metabolic characteristics, or cell differentiation states. More accurately, UCP2 functions as a buffer or amplifier within the therapeutic tolerance network. Therefore, determining whether UCP2 serves as a driving factor or merely an associated marker is the initial task that must be addressed when considering its use as a therapeutic target or associated marker (9, 14, 41).

## 5 UCP2 and the tumor immune microenvironment

### 5.1 Role of UCP2 in T-cell metabolic reprogramming

**Figure 2:**
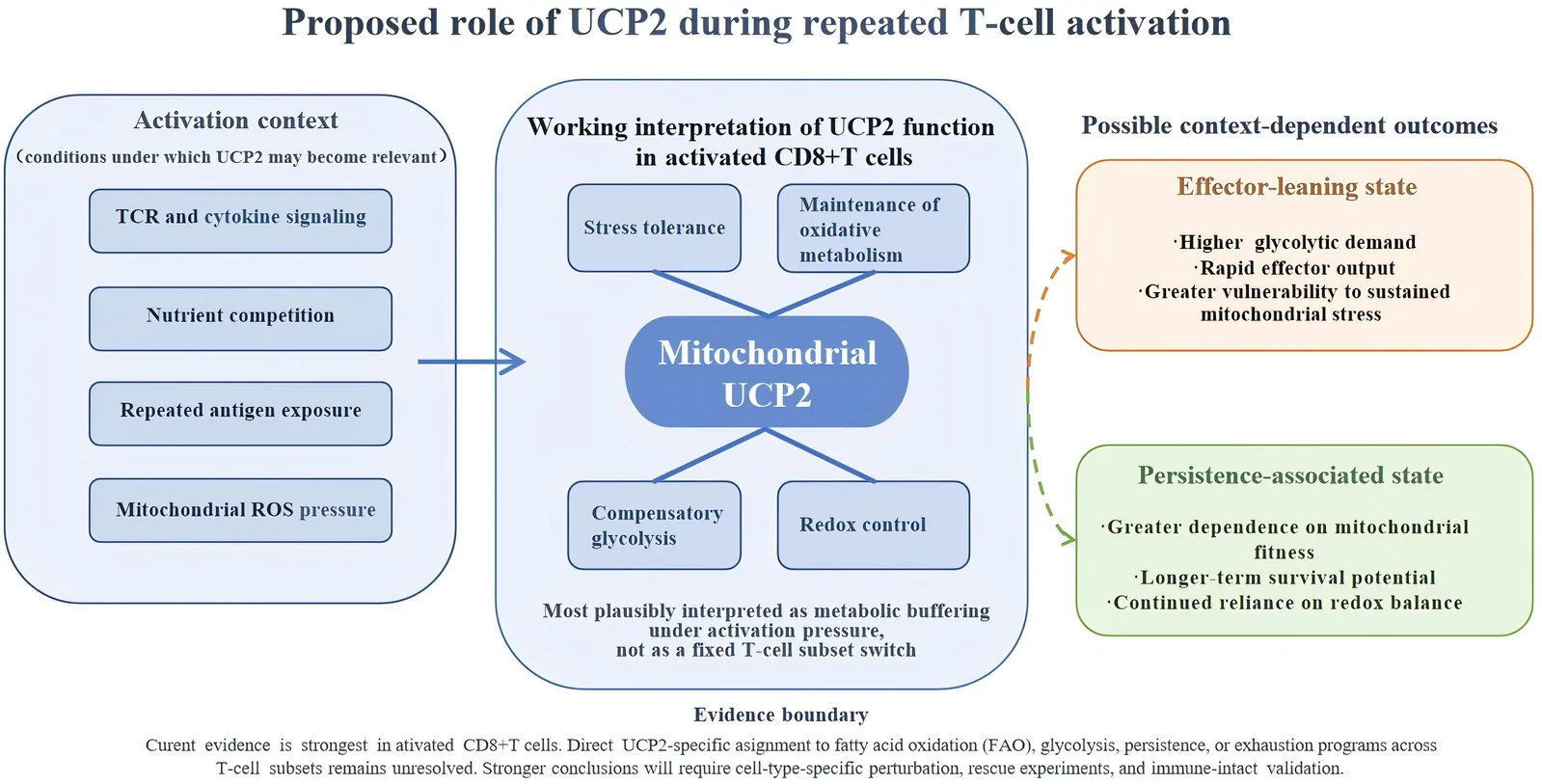
Proposed role of UCP2 during repeated T-cell activation. Current evidence is strongest in activated CD8+ T cells, in which UCP2 is most plausibly interpreted as a regulator of metabolic buffering under repeated activation pressure, including stress tolerance, maintenance of oxidative metabolism, compensatory glycolysis, and redox control. The schematic highlights possible context-dependent bias toward effector-leaning or persistence-associated states, but direct UCP2-specific assignment to fatty acid oxidation (FAO), glycolysis, persistence, or exhaustion programs across T-cell subsets remains unresolved.

Interest in UCP2 within the tumor immune microenvironment has arisen primarily from T-cell studies rather than from a broad, equally mature literature across all stromal or myeloid compartments. In antigen-stimulated CD8+ T-cell systems, UCP2 has been implicated in post-activation metabolic reprogramming, particularly in the balance among mitochondrial oxidative metabolism, glycolysis, and ROS signaling (30, 31) (**Figure 2**). This is a narrower and more defensible conclusion than simply labeling UCP2 as pro-immunity or anti-immunity. A more careful interpretation is that UCP2 helps set the metabolic stress threshold encountered during repeated activation, so its effect is likely to vary with nutrient availability, activation history, and the oxidative burden imposed by the local tumor environment.

Tumor-context evidence adds translational relevance, but it also adds interpretive complexity. In PDAC, dual targeting of UCP2 and IL-17 signaling altered T-cell metabolism, enhanced IFN-gamma production and cytotoxic programs, and improved antitumor activity in preclinical models (15). However, that result emerges from a multicellular inflammatory setting and does not by itself establish whether the dominant driver is tumor-cell UCP2, T-cell UCP2, or the interaction between both compartments. We therefore treat the T-cell literature as the strongest direct entry point into immune-metabolic UCP2 biology, while considering whole-tumor response phenotypes as integrative but less cell-type-resolved evidence.

### 5.2 Potential effects of UCP2 on CD8+ T-cell effector function and exhaustion

Taken together, these data support the cautious view that UCP2 may modulate the balance between effector function and persistence, but do not yet justify a direct causal claim in exhaustion biology.

At the mechanistic level, the current evidence is more consistent with a metabolic stress-tuning model than with a simple pathway switch controlled uniformly by UCP2. In activated CD8+ T cells, UCP2 appears most relevant where mitochondrial oxidative metabolism, compensatory glycolysis, and ROS tolerance must be balanced during repeated stimulation (30, 31). In short-lived effector settings, excessive mitochondrial stress may impair expansion or cytokine production, whereas in longer-lived persistence-associated states, preservation of mitochondrial fitness may become more important than maximal glycolytic drive. This does not yet justify a universal statement that UCP2 drives fatty acid oxidation in one subset and glycolysis in another; rather, it suggests that UCP2 may influence how T cells move along this metabolic continuum under activation pressure. Direct evidence remains strongest in activated CD8+ T cells, whereas subset-specific assignments across memory-like, exhausted, regulatory, or other T-cell states remain limited and should be interpreted cautiously.

The most prudent conclusion at present is that UCP2 may modulate the trade-off between CD8+ T cell effector output and long-term persistence. Definitive demonstration of this dual role will require T cell-specific UCP2 deletion, rescue experiments, longitudinal assessment of exhaustion markers, and immunocompetent tumor models. UCP2-targeted immunotherapy cannot be approached with the same logic used for tumor-cell-intrinsic targets. Inhibiting UCP2 in malignant cells may elevate ROS and disrupt metabolic adaptation, whereas the same intervention in CD8+ T cells could compromise mitochondrial fitness or shorten effector persistence. Dose, timing, delivery route, and choice of combination partner will therefore be critical; if these variables are neglected, tumor-cell benefits and immune-cell costs may offset one another (15, 31, 32).

### 5.3 Compartment-specific roles of UCP2 in tumor and immune cells

The same UCP2 perturbation cannot be assumed to mean the same thing in malignant cells and immune cells. In tumor cells, the most reproducible data connect UCP2 to mitochondrial homeostasis, attenuation of damaging ROS excursions, and maintenance of carbon flow into anabolic and redox-supporting pathways under nutrient or treatment stress (6, 10, 20). In activated T cells, by contrast, the clearest evidence concerns metabolic adaptation after stimulation, including redox control, mitochondrial fitness, and the balance between oxidative metabolism and compensatory glycolysis (15, 30, 31). For this reason, cross-cell-type comparisons are most informative when they are made within the same biological model rather than across unrelated cancer systems.

This compartmental distinction is also essential for therapeutic interpretation. Systemic or prolonged UCP2 inhibition could deprive tumor cells of a stress-adaptive advantage while at the same time weakening effector immune-cell fitness. Conversely, a tumor in which the dominant UCP2-dependent phenotype resides in malignant cells may still benefit from carefully timed or locally delivered inhibition. The practical question is therefore not whether UCP2 is uniformly beneficial or harmful, but which compartment is more functionally dependent on UCP2 in a given setting and whether an intervention can separate tumor-cell benefit from immune-cell cost.

### 5.4 Direct and integrative evidence for UCP2 in the immune microenvironment

Within the immune microenvironment, the strongest current support concerns T-cell metabolism and whole-tumor immune-cycle phenotypes rather than macrophage or fibroblast programs. Antigen-stimulated T-cell studies provide the most direct evidence that UCP2 can influence post-activation metabolic programming (30, 31). By contrast, studies linking UCP2 to dendritic-cell recruitment, CD8+ T-cell infiltration, or vascular normalization generally derive from integrated tumor models in which multiple cell populations are being remodeled simultaneously (32).

This hierarchy of evidence matters because whole-tumor immune readouts can be biologically meaningful without being cell-type-specific. A change in recruitment or infiltration may reflect altered tumor-cell metabolism, cytokine signaling, stromal tone, vascular function, or a combination of these processes. We therefore regard UCP2 as a metabolism-immune bridge only where the relevant compartment and mechanism are reasonably defined; otherwise, the more accurate description is that UCP2-associated metabolic states correlate with immune-response phenotypes that still require cell-type-resolved testing.

### 5.5 Candidate roles beyond T cells: myeloid, endothelial, and fibroblast contexts

The immune-microenvironment discussion should not stop at T cells, because macrophages, myeloid-derived suppressor cells, endothelial cells, and cancer-associated fibroblast niches all operate under substantial mitochondrial, redox, and nutrient constraints. In principle, a carrier that modulates mitochondrial stress, ROS pressure, or C4 metabolite exchange could influence macrophage polarization, myeloid suppressive function, endothelial stress adaptation, or fibroblast-conditioned inflammatory circuits. Inflammatory programs such as IL-17-associated signaling or neutrophil-dominated remodeling in PDAC may further shape how UCP2-targeted interventions are expressed, even if UCP2 itself has not yet been mapped directly in each compartment (15, 42–44).

At present, however, these roles remain largely inferential. The literature does not yet provide the same level of direct, cell-type-specific evidence for TAMs, MDSCs, endothelial cells, or fibroblast-linked programs as it does for activated T cells or for several tumor-cell metabolic settings. These contexts should therefore be framed as hypotheses that deserve testing rather than as established mechanisms. Progress will require single-cell or spatially resolved mapping, perturbation in defined stromal or immune compartments, and validation in immunocompetent models that preserve the metabolic competition of the native microenvironment (33–35).

## 6 The clinical value of UCP2: biomarkers and therapeutic targets

### 6.1 Potential and practical boundaries as a biomarker

From a translational perspective, UCP2 is better regarded as a candidate indicator than as a mature biomarker. The principal barriers are well known but critical: no prospective validation, no standardized assay, and no consensus on functional interpretation. Current studies employ IHC, qPCR, Western blotting, or public mRNA datasets, but these readouts capture distinct aspects of biology. mRNA levels do not reliably predict protein abundance, and protein abundance does not establish correct localization or transport activity. IHC scoring further depends on antibody specificity, tissue processing, and reader criteria. A useful UCP2 biomarker strategy therefore must link mRNA, protein, and function, ideally incorporating OCR/ECAR, baseline ROS, glutamine dependence, NADPH/GSH status, and immune infiltration (9, 11, 13, 41, 45). Compared with established metabolic markers such as IDH mutations or lactate dehydrogenase (LDH), UCP2 is less ready for clinical application. IDH mutation represents a defined genetic event with standardized detection and clear therapeutic implications, whereas UCP2 reflects a dynamic metabolic state shaped by nutrition, hypoxia, inflammation, and treatment pressure. At present, UCP2 is unlikely to serve as a standalone marker; a more realistic role is within a stratification model that integrates cancer type, driver mutation, metabolic dependence, and immune status. Clinical trial evidence to support such a model is still lacking.

For clinical validation, tissue-based assessment is currently more realistic than plasma- or circulating-tumor-cell-based approaches because UCP2 interpretation depends not only on abundance but also on mitochondrial localization, tumor-cell versus stromal/immune-cell signal, and metabolic context. An initial biomarker strategy should therefore prioritize formalin-fixed paraffin-embedded tumor tissue using multiplex immunohistochemistry or immunofluorescence with automated or semi-automated scoring, ideally combined with markers of proliferation, mitochondrial state, and immune infiltration. Among the settings discussed in this review, PDAC and selected hematologic malignancies may be among the more informative initial contexts because UCP2-linked glutamine dependence, redox control, and treatment adaptation are more mechanistically developed there than in many other tumor types. Minimum validation requirements should include an independent external cohort, multivariate analysis against established clinicopathological variables, and, where feasible, prospective evaluation. Any clinically useful UCP2 biomarker should also be compared against existing disease-specific markers rather than being proposed as a stand-alone readout.

### 6.2 Therapeutic appeal: mechanistic rationale and development challenges

UCP2 is an attractive therapeutic target because it links mitochondrial metabolism, redox regulation, and stress adaptation. In AML, PDAC, ULMS, melanoma, and select NSCLC models, modulation of UCP2 activity shifts ROS thresholds, anaplerosis, and pro-survival signaling, producing context-dependent effects on proliferation and treatment response (10, 20, 23, 24, 27, 46, 47). Potential influences on the tumor microenvironment and T-cell metabolism further expand the therapeutic implications beyond tumor-cell-intrinsic biology (15, 32). Targeting UCP2 nevertheless poses practical challenges. The protein resides in the inner mitochondrial membrane, so any drug must traverse multiple biological barriers to reach its site of action, and assessing target occupancy or conformational selectivity in that compartment remains difficult. Mitochondrial delivery strategies, including TPP+ conjugation, mitochondria-penetrating peptides, and nanocarriers, can increase mitochondrial enrichment but do not by themselves resolve tumor selectivity, normal-tissue toxicity, pharmacokinetics, or immune-cell liabilities (48). A further limitation is pharmacological specificity. A high-resolution structural framework that distinguishes orthosteric inhibition, allosteric regulation, and substrate competition for UCP2 is not yet available. Genipin is therefore best treated as a proof-of-concept tool rather than as a clean mechanistic or clinical-grade inhibitor, and its interpretive limitations warrant explicit caution when UCP2-dependent phenotypes are assigned (37–39, 46, 47).

A more plausible development strategy is to begin by defining settings in which UCP2 dependence is biologically supported and only then align delivery, pharmacology, and combination design with that context. In practice, this means integrating tumor-targeted delivery, prodrug or nanoparticle approaches, and cell-type-specific genetic models to widen the therapeutic window between tumor-cell inhibition and preservation of effector immune-cell function (15, 33–35, 48). Under this view, UCP2 is less compelling as a target for broad inhibitor-first development and more useful as a context-linked metabolic vulnerability that requires dependency mapping before compound optimization.

### 6.3 Current intervention strategies: tool compounds, genetic validation, and combination therapy

Current interventions targeting UCP2 remain at the preclinical stage. Their main value lies less in offering clinically ready inhibitors than in exposing context-specific vulnerabilities and sensitization strategies. This point is especially important for genipin. Across PDAC, HER2-positive breast cancer, gallbladder cancer, and glioblastoma models, genipin has been used to reproduce ROS-centered, mTOR-linked, or drug-sensitizing phenotypes associated with UCP2 perturbation (37–39, 46, 47). Yet genipin should be treated as a proof-of-concept tool rather than as a clean mechanistic probe, because the resulting phenotypes are difficult to separate from broader effects on mitochondrial stress, redox balance, and downstream cell-death pathways.

Accordingly, genipin-based conclusions are strongest when they are supported by orthogonal evidence, such as genetic knockdown or knockout, rescue experiments, metabolic flux measurements, or an independent perturbation strategy. Without such controls, findings involving apoptosis, autophagy, ferroptosis, or chemosensitization should be interpreted as UCP2-associated rather than as definitive consequences of selective UCP2 inhibition (37–39, 46, 47). The translational implication is therefore not that genipin itself is a development candidate, but that UCP2-linked dependencies may identify combination settings worth testing more rigorously.

From that perspective, PDAC remains one of the better-supported contexts because KRAS-linked glutamine-aspartate metabolism, ROS-GAPDH biology, and mTOR-related crosstalk provide a coherent rationale for pairing UCP2 perturbation with gemcitabine or pathway-directed combinations (10, 21, 46, 47). HER2-positive breast cancer, gallbladder cancer, and glioblastoma represent earlier-stage contexts in which modulation of UCP2 may influence response to trastuzumab, gemcitabine-based therapy, or ferroptosis-oriented strategies, but substantially stronger *in vivo*, PDO/PDX, or organoid validation is still needed (37–39). A 2025 HCC study on OSW-1 provides a non-genipin example, suggesting that UCP2-directed pharmacology may extend beyond the existing genipin literature, although selectivity and target-engagement questions remain unresolved there as well (49). Immunotherapy-oriented combinations, including IL-17 blockade or checkpoint-based strategies in PDAC, remain intriguing but technically more difficult because tumor-cell and T-cell effects must be disentangled in immune-intact models (15, 32, 50).

### 6.4 From pan-cancer target to context-dependent metabolic vulnerability

**Figure 3:**
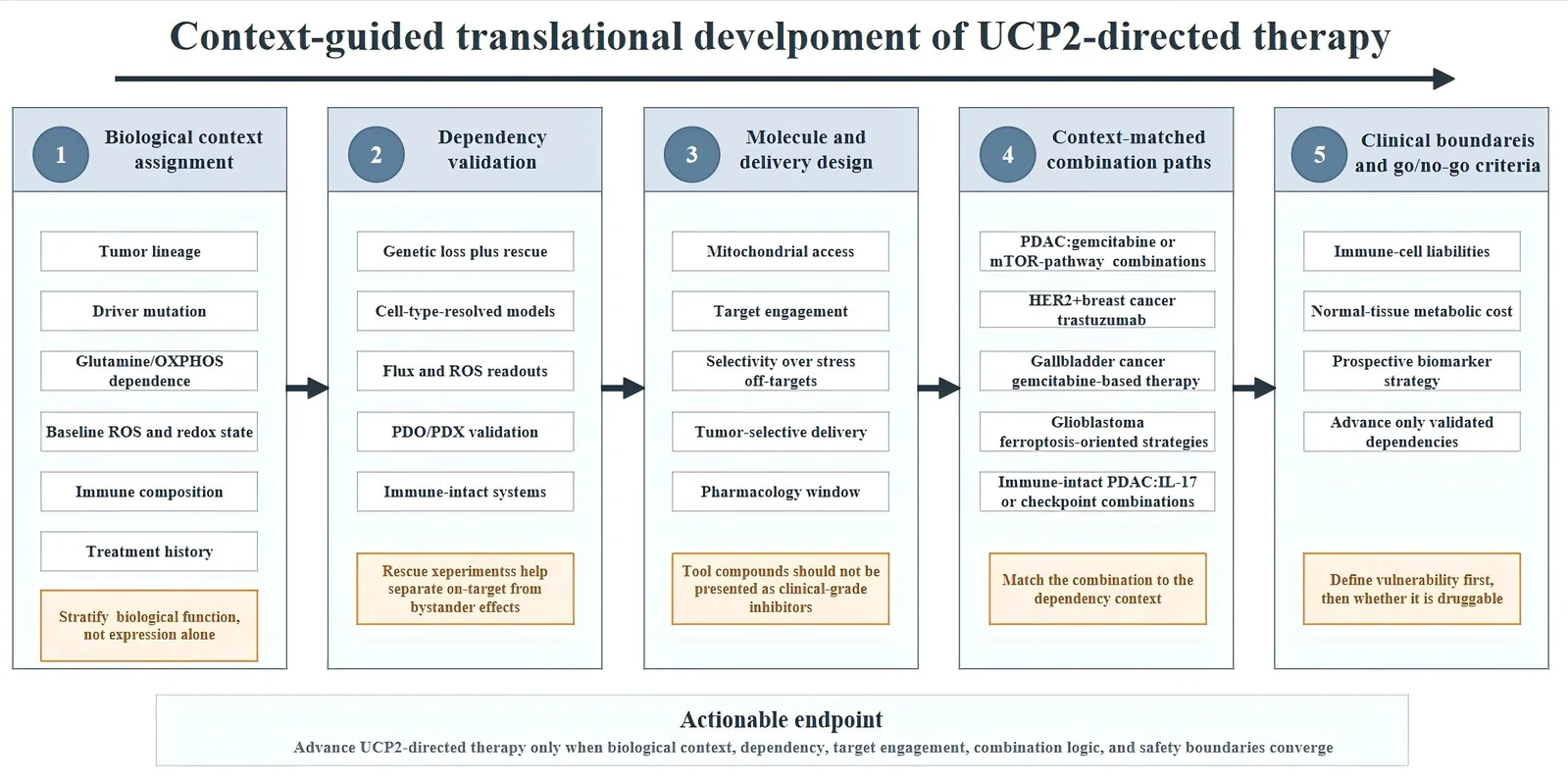
Context-guided translational development of UCP2-directed therapy. The schematic emphasizes that development should begin with biological context assignment, including tumor lineage, driver mutation, metabolic dependency, baseline redox state, immune composition, and treatment history. Dependency validation then requires genetic loss-plus-rescue experiments, cell-type-resolved models, and flux or ROS readouts in PDO, PDX, or immune-intact systems. Molecule development must establish mitochondrial access, target engagement, and selectivity, whereas combinations should be matched to specific dependency contexts rather than applied generically. Overall, the framework supports the view that UCP2 is more useful as a stratified metabolic vulnerability than as a universal pan-cancer target.

The central translational point is that UCP2 is unlikely to be useful as a universal target across all cancers. A more practical designation is context-dependent metabolic vulnerability, in which patient selection is based on tumor lineage, driver mutation, nutrient dependence, redox state, and immune context rather than on UCP2 expression alone. In some tumors, UCP2 appears to support mitochondrial homeostasis, ROS buffering, and anaplerosis; in others, it may mainly reflect mitochondrial state or immune-metabolic composition. In still other settings, the dominant biology may reside in the microenvironment rather than in malignant cells themselves (6, 7, 12, 15, 32). This interpretation supports a development path that begins with biologically defined UCP2-dependent settings and only then advances to drug design and rational combination testing (**Figure 3**).

## 7 Conclusions and perspectives

UCP2 resists a simple pro-tumor versus anti-tumor classification because its consequences depend on driver background, substrate availability, redox pressure, and immune context. Across studies, UCP2 has been associated with control of membrane potential, ROS handling, C4 metabolite transport, glutamine anaplerosis, mTOR/HIF-related signaling, and immune-cell metabolic adaptation (6–11, 15, 16, 30, 31). The most consistent interpretation is not that UCP2 exerts one fixed action across cancers, but that it functions as a conditional mitochondrial regulator whose importance rises in defined biological contexts. Future translational work should therefore stratify tumors by lineage, mutation, metabolic dependence, and immune state before treating UCP2 as a biomarker or therapeutic target.

Several priorities follow from this view. First, UCP2 studies require clearer stratification by cancer type, driver mutation, mitochondrial dependence, and immune state. Second, the field needs improved tools, including more selective modulators, cell-type-specific genetic models, rescue experiments, and reproducible metabolic readouts, to distinguish true UCP2 dependence from a nonspecific marker of mitochondrial stress (15, 32, 33). Third, immune-intact validation remains important because UCP2 may exert divergent effects in tumor cells and immune cells (15, 32). Finally, current evidence suggests that UCP2 is more likely to be useful as part of rational combination therapy than as a standalone drug target. The most informative next studies will therefore test chemotherapy, metabolic therapy, radiotherapy, or immunomodulation in models in which UCP2 dependence has already been defined. Overall, UCP2 is best viewed as a context-dependent metabolic vulnerability rather than as a universal anticancer target.

## Glossary

ACSL4: acyl-CoA synthetase long-chain family member 4
ADP: adenosine diphosphate
AKT: protein kinase B
AML: acute myeloid leukemia
ATP: adenosine triphosphate
DDX5: DEAD-box helicase 5
ECAR: extracellular acidification rate
ETC: electron transport chain
FAO: fatty acid oxidation
GOT1: glutamic-oxaloacetic transaminase 1
GPX4: glutathione peroxidase 4
HCC: hepatocellular carcinoma
HER2: human epidermal growth factor receptor 2
HIF-1alpha: hypoxia-inducible factor 1-alpha
IDH: isocitrate dehydrogenase
IFN-gamma: interferon-gamma
IHC: immunohistochemistry
IL-17: interleukin-17
KRAS: Kirsten rat sarcoma viral oncogene homolog
LDH: lactate dehydrogenase
LKB1/STK11: liver kinase B1/serine/threonine kinase 11
MDH1: malate dehydrogenase 1
MDSCs: myeloid-derived suppressor cells
ME1: malic enzyme 1
mTOR: mechanistic target of rapamycin
NF-kappaB: nuclear factor kappa B
NSCLC: non-small cell lung cancer
OCR: oxygen consumption rate
PDAC: pancreatic ductal adenocarcinoma
PDO: patient-derived organoid
PDX: patient-derived xenograft
PI3K: phosphoinositide 3-kinase
PPAR-gamma: peroxisome proliferator-activated receptor gamma
ROS: reactive oxygen species
TAMs: tumor-associated macrophages
SLC25: solute carrier family 25
T-ALL: T-cell acute lymphoblastic leukemia
TCA: tricarboxylic acid
TPP+: triphenylphosphonium
UBAP2L: ubiquitin-associated protein 2-like
UCP2: uncoupling protein 2
ULMS: uterine leiomyosarcoma

## References

[1] Hanahan D Weinberg RA . Hallmarks of cancer: the next generation. Cell. (2011) 144:646–74. doi: 10.1016/j.cell.2011.02.013 21376230

[2] Martinez-Reyes I Chandel NS . Cancer metabolism: looking forward. Nat Rev Cancer. (2021) 21:669–80. doi: 10.1038/s41568-021-00378-6 34272515

[3] Vyas S Zaganjor E Haigis MC . Mitochondria and cancer. Cell. (2016) 166:555–66. doi: 10.1016/j.cell.2016.07.002 27471965 PMC5036969

[4] Zong WX Rabinowitz JD White E . Mitochondria and cancer. Mol Cell. (2016) 61:667–76. doi: 10.1016/j.molcel.2016.02.011 26942671 PMC4779192

[5] Musicco C Signorile A Pesce V Loguercio Polosa P Cormio A . Mitochondria deregulations in cancer offer several potential targets of therapeutic interventions. Int J Mol Sci. (2023) 24:10420. doi: 10.3390/ijms241310420 37445598 PMC10342097

[6] Luby A Alves-Guerra MC . UCP2 as a cancer target through energy metabolism and oxidative stress control. Int J Mol Sci. (2022) 23:15077. doi: 10.3390/ijms232315077 36499405 PMC9735768

[7] Nesci S Rubattu S . UCP2, a member of the mitochondrial uncoupling proteins: an overview from physiological to pathological roles. Biomedicines. (2024) 12:1307. doi: 10.3390/biomedicines12061307 38927514 PMC11201685

[8] Hirschenson J Melgar-Bermudez E Mailloux RJ . The uncoupling proteins: a systematic review on the mechanism used in the prevention of oxidative stress. Antioxidants (Basel). (2022) 11:322. doi: 10.3390/antiox11020322 35204205 PMC8868465

[9] Beikbaghban T Proietti L Ebner J Sango R Rattei T Weichhart T . Differential regulation of mitochondrial uncoupling protein 2 in cancer cells. Biochim Biophys Acta Bioenerg. (2024) 1865:149486. doi: 10.1016/j.bbabio.2024.149486 38986826 PMC7617651

[10] Raho S Capobianco L Malivindi R Vozza A Piazzolla C De Leonardis F . KRAS-regulated glutamine metabolism requires UCP2-mediated aspartate transport to support pancreatic cancer growth. Nat Metab. (2020) 2:1373–81. doi: 10.1038/s42255-020-00315-1 33230296

[11] Vozza A Parisi G De Leonardis F Lasorsa FM Castegna A Amorese D . UCP2 transports C4 metabolites out of mitochondria, regulating glucose and glutamine oxidation. Proc Natl Acad Sci USA. (2014) 111:960–5. doi: 10.1073/pnas.1317400111 24395786 PMC3903233

[12] Yu X Shi M Wu Q Wei W Sun S Zhu S . Identification of UCP1 and UCP2 as potential prognostic markers in breast cancer: a study based on immunohistochemical analysis and bioinformatics. Front Cell Dev Biol. (2022) 10:891731. doi: 10.3389/fcell.2022.891731 35874806 PMC9300932

[13] Segales J Sanchez-Martin C Pujol-Morcillo A Martin-Ruiz M de Los Santos P Lobato-Alonso D . Role of UCP2 in the energy metabolism of the cancer cell line A549. Int J Mol Sci. (2023) 24:8123. doi: 10.3390/ijms24098123 37175829 PMC10179244

[14] Guo W Chen J Li S Zeng X Wu X . Integrative pan-cancer analysis of UCP family and experimental validation identifies UCP2 as a potential therapeutic target for glioma. Front Cell Dev Biol. (2025) 13:1662654. doi: 10.3389/fcell.2025.1662654 41403699 PMC12702733

[15] Liu C Yeh C Wu T Lin C Kuo Y Iwakura Y . Metabolic reprogramming of T cells by dual UCP2 and IL-17 blockade enhances immunity against pancreatic cancer. Adv Sci (Weinh). (2026) 13:e13020. doi: 10.1002/advs.202513020 41486584 PMC13042516

[16] Sancerni T Renoult O Luby A Caradeuc C Lenoir V Croyal M . UCP2 silencing restrains leukemia cell proliferation through glutamine metabolic remodeling. Front Immunol. (2022) 13:960226. doi: 10.3389/fimmu.2022.960226 36275699 PMC9582289

[17] Alden RS Kamran MZ Bashjawish BA Simone BA . Glutamine metabolism and radiosensitivity: beyond the Warburg effect. Front Oncol. (2022) 12:1070514. doi: 10.3389/fonc.2022.1070514 36465373 PMC9712788

[18] Kang Y Chen L . Structural basis for the binding of DNP and purine nucleotides onto UCP1. Nature. (2023) 620:226–31. doi: 10.1038/s41586-023-06332-w 37336486

[19] Ruprecht JJ King MS Zogg T Aleksandrova AA Pardon E Crichton PG . The molecular mechanism of transport by the mitochondrial ADP/ATP carrier. Cell. (2019) 176:435–47.e15. doi: 10.1016/j.cell.2018.11.025 30611538 PMC6349463

[20] Innocent AO Shen Y Gao Y Sun R Aximujiang K Xu Z . Suppression of UCP2 alleviates leukemogenesis by enhancing branched-chain amino acids-induced oxidative stress via activating the PI3K/AKT/mTOR signaling pathway. Genes Dis. (2026) 13:101794. doi: 10.1016/j.gendis.2025.101794 42004221 PMC13089170

[21] Caggiano EG Taniguchi CM . UCP2 and pancreatic cancer: conscious uncoupling for therapeutic effect. Cancer Metastasis Rev. (2024) 43:777–94. doi: 10.1007/s10555-023-10157-4 38194152 PMC11156755

[22] Wang K Zhang L Deng B Zhao K Chen C Wang W . Mitochondrial uncoupling protein 2: a central player in pancreatic disease pathophysiology. Mol Med. (2024) 30:259. doi: 10.1186/s10020-024-01027-y 39707176 PMC11660649

[23] Nagao Y Yokoi A Yoshida K Sugiyama M Watanabe E Nakamura K . Novel therapeutic strategies targeting UCP2 in uterine leiomyosarcoma. Pharmacol Res. (2023) 189:106693. doi: 10.1016/j.phrs.2023.106693 36773710

[24] Li J Jia Y An L Niu C Cong X Zhao Y . Uncoupling protein 2 is upregulated in melanoma cells and contributes to the activation of Akt/mTOR and ERK signaling. Int J Oncol. (2020) 56:1252–61. doi: 10.3892/ijo.2020.5010 32319575

[25] Ge Z Shang Y Wang W Yang J Chen SZ . Brown adipocytes promote epithelial mesenchymal transition of neuroblastoma cells by inducing PPAR-gamma/UCP2 expression. Adipocyte. (2022) 11:335–45. doi: 10.1080/21623945.2022.2073804 35531888 PMC9122313

[26] Bansal M Gupta A Ding HF . MYCN and metabolic reprogramming in neuroblastoma. Cancers (Basel). (2022) 14:4113. doi: 10.3390/cancers14174113 36077650 PMC9455056

[27] Song C Liu Q Qin J Liu L Zhou Z Yang H . UCP2 promotes NSCLC proliferation and glycolysis via the mTOR/HIF-1alpha signaling. Cancer Med. (2024) 13:e6938. doi: 10.1002/cam4.6938 38217303 PMC10905227

[28] Yang Z Li G Zhao Y Zhang L Yuan X Meng L . Molecular insights into the recruiting between UCP2 and DDX5/UBAP2L in the metabolic plasticity of non-small-cell lung cancer. J Chem Inf Model. (2021) 61:3978–87. doi: 10.1021/acs.jcim.1c00138 34308648

[29] Angelopoulou A Theocharous G Valakos D Polyzou A Magkouta S Myrianthopoulos V . Loss of the tumour suppressor LKB1/STK11 uncovers a leptin-mediated sensitivity mechanism to mitochondrial uncouplers for targeted cancer therapy. Mol Cancer. (2024) 23:147. doi: 10.1186/s12943-024-02061-4 39048991 PMC11270803

[30] Sukumar M Cheng K Gangaplara A Patel Y Vodnala SK Islam R . Integrated computational and experimental analysis identifies the mitochondrial uncoupling protein 2 (Ucp2) as a key regulator of T cell antitumor function [abstract. Cancer Res. (2021) 81:Abstract nr 1527. doi: 10.1158/1538-7445.AM2021-1527 36230740

[31] Chaudhuri L Srivastava RK Kos F Shrikant PA . Uncoupling protein 2 regulates metabolic reprogramming and fate of antigen-stimulated CD8+ T cells. Cancer Immunol Immunother. (2016) 65:869–74. doi: 10.1007/s00262-016-1851-4 27271549 PMC4919150

[32] Cheng WC Tsui YC Ragusa S Koelzer VH Mina M Franco F . Uncoupling protein 2 reprograms the tumor microenvironment to support the anti-tumor immune cycle. Nat Immunol. (2019) 20:206–17. doi: 10.1038/s41590-018-0290-0 30664764

[33] Cao Y Li X Pan Y Wang H Yang S Hong L . CRISPR-based genetic screens advance cancer immunology. Sci China Life Sci. (2024) 67:2554–62. doi: 10.1007/s11427-023-2571-0 39048715

[34] Ma X Fernandez FM . Advances in mass spectrometry imaging for spatial cancer metabolomics. Mass Spectrom Rev. (2024) 43:235–68. doi: 10.1002/mas.21804 36065601 PMC9986357

[35] Liu X Peng T Xu M Lin S Hu B Chu T . Spatial multi-omics: deciphering technological landscape of integration of multi-omics and its applications. J Hematol Oncol. (2024) 17:72. doi: 10.1186/s13045-024-01596-9 39182134 PMC11344930

[36] Gao T Yang L Zhang Y Bajinka O Yuan X . Cancer metabolic reprogramming and precision medicine-current perspective. Front Pharmacol. (2024) 15:1450441. doi: 10.3389/fphar.2024.1450441 39484162 PMC11524845

[37] Hua J Zhang Z Zhang L Sun Y Yuan Y . UCP-2 inhibitor enhanced the efficacy of trastuzumab against HER2-positive breast cancer cells. Cancer Chemother Pharmacol. (2021) 88:633–42. doi: 10.1007/s00280-021-04303-4 34146128 PMC8367901

[38] Yu J Shi L Lin W Lu B Zhao Y . UCP2 promotes proliferation and chemoresistance through regulating the NF-kappaB/beta-catenin axis and mitochondrial ROS in gallbladder cancer. Biochem Pharmacol. (2020) 172:113745. doi: 10.1016/j.bcp.2019.113745 31811866 PMC6981058

[39] Dong H Sun K Wang X Cui M Ma Y Li K . Repurposed genipin targeting UCP2 exhibits antitumor activity through inducing ferroptosis in glioblastoma. Acta Biochim Biophys Sin (Shanghai). (2025) 57:403–14. doi: 10.3724/abbs.2024168 39523775 PMC11986454

[40] Dixon SJ Lemberg KM Lamprecht MR Skouta R Zaitsev EM Gleason CE . Ferroptosis: an iron-dependent form of nonapoptotic cell death. Cell. (2012) 149:1060–72. doi: 10.1016/j.cell.2012.03.042 22632970 PMC3367386

[41] Vallejo FA Vanni S Graham RM . UCP2 as a potential biomarker for adjunctive metabolic therapies in tumor management. Front Oncol. (2021) 11:640720. doi: 10.3389/fonc.2021.640720 33763373 PMC7982524

[42] Liu W Wang X Wu W . Role and functional mechanisms of IL-17/IL-17R signaling in pancreatic cancer. Oncol Rep. (2024) 52:144. doi: 10.3892/or.2024.8803 39219271 PMC11378154

[43] Chen Z Qiao S Yang L Sun M Li B Lu A . Mechanistic insights into the roles of the IL-17/IL-17R families in pancreatic cancer. Int J Mol Sci. (2023) 24:13539. doi: 10.3390/ijms241713539 37686343 PMC10487659

[44] Steele CW Karim SA Leach JDG Bailey P Upstill-Goddard R Rishi L . CXCR2 inhibition profoundly suppresses metastases and augments immunotherapy in pancreatic ductal adenocarcinoma. Cancer Cell. (2016) 29:832–45. doi: 10.1016/j.ccell.2016.04.014 27265504 PMC4912354

[45] Bucksot J Ritchie K Biancalana M Cole JA Cook D . Pan-cancer, genome-scale metabolic network analysis of over 10,000 patients elucidates relationship between metabolism and survival. Cancers (Basel). (2024) 16:2302. doi: 10.3390/cancers16132302 39001365 PMC11240338

[46] Dando I Fiorini C Pozza ED Padroni C Costanzo C Palmieri M . UCP2 inhibition triggers ROS-dependent nuclear translocation of GAPDH and autophagic cell death in pancreatic adenocarcinoma cells. Biochim Biophys Acta. (2013) 1833:672–9. doi: 10.1016/j.bbamcr.2012.10.028 23124112

[47] Dando I Pacchiana R Pozza ED Cataldo I Bruno S Conti P . UCP2 inhibition induces ROS/AKT/mTOR axis: role of GAPDH nuclear translocation in genipin/everolimus anticancer synergism. Free Radic Biol Med. (2017) 113:176–89. doi: 10.1016/j.freeradbiomed.2017.09.022 28962872

[48] Batheja S Gupta S Tejavath KK Gupta U . TPP-based conjugates: potential targeting ligands. Drug Discov Today. (2024) 29:103983. doi: 10.1016/j.drudis.2024.103983 38641237

[49] Shen K Jin X . OSW-1 inhibits tumor cell proliferation and migration via uncoupling protein 2 in hepatocellular carcinoma. Oncol Lett. (2025) 30:361. doi: 10.3892/ol.2025.15107 40469918 PMC12134977

[50] Kolb D Kolishetti N Surnar B Sarkar S Guin S Shah AS . Metabolic modulation of the tumor microenvironment leads to multiple checkpoint inhibition and immune cell infiltration. ACS Nano. (2020) 14:11055–66. doi: 10.1021/acsnano.9b10037 32706241

